# Molecular Nutrition Research—The Modern Way Of Performing Nutritional Science

**DOI:** 10.3390/nu4121898

**Published:** 2012-12-03

**Authors:** Frode Norheim, Ingrid M. F. Gjelstad, Marit Hjorth, Kathrine J. Vinknes, Torgrim M. Langleite, Torgeir Holen, Jørgen Jensen, Knut Tomas Dalen, Anette S. Karlsen, Anders Kielland, Arild C. Rustan, Christian A. Drevon

**Affiliations:** 1 Department of Nutrition, Institute of Basic Medical Sciences, Faculty of Medicine, University of Oslo, P.O. Box 1046, Blindern, N-0317 Oslo, Norway; Email: frode.norheim@medisin.uio.no (F.N.); i.m.f.gjelstad@medisin.uio.no (I.M.F.G.); marit.hjorth@medisin.uio.no (M.H.); kathrine.vinknes@medisin.uio.no (K.J.V.); t.m.langleite@medisin.uio.no (T.M.L.); torgeir.holen@medisin.uio.no (T.H.); k.t.dalen@medisin.uio.no (K.T.D.); anette.karlsen@medisin.uio.no (A.S.K.); anders.kielland@medisin.uio.no (A.K.); 2 Department of Physical Performance, Norwegian School of Sport Science, P.O. Box 4014, Ullevål Stadion, N-0806 Oslo, Norway; Email: Jorgen.Jensen@nih.no; 3 Department of Pharmaceutical Biosciences, School of Pharmacy, University of Oslo, P.O. Box 1068, Blindern, N-0316 Oslo, Norway; Email: arild.rustan@farmasi.uio.no

**Keywords:** molecular nutrition, nutrigenomics, genomics, transcriptomics, proteomics, metabolomics, systems biology, adipokines, myokines

## Abstract

In spite of amazing progress in food supply and nutritional science, and a striking increase in life expectancy of approximately 2.5 months per year in many countries during the previous 150 years, modern nutritional research has a great potential of still contributing to improved health for future generations, granted that the revolutions in molecular and systems technologies are applied to nutritional questions. Descriptive and mechanistic studies using state of the art epidemiology, food intake registration, genomics with single nucleotide polymorphisms (SNPs) and epigenomics, transcriptomics, proteomics, metabolomics, advanced biostatistics, imaging, calorimetry, cell biology, challenge tests (meals, exercise, *etc.*), and integration of all data by systems biology, will provide insight on a much higher level than today in a field we may name molecular nutrition research. To take advantage of all the new technologies scientists should develop international collaboration and gather data in large open access databases like the suggested Nutritional Phenotype database (dbNP). This collaboration will promote standardization of procedures (SOP), and provide a possibility to use collected data in future research projects. The ultimate goals of future nutritional research are to understand the detailed mechanisms of action for how nutrients/foods interact with the body and thereby enhance health and treat diet-related diseases.

## 1. Introduction

Mankind has gone through several revolutions concerning dietary habits. A large break-through was the mastering of fire to be able to cook or fry foods making nutrients more bioavailable in particular to children. This more systematic use of fire related to butchering took place in several places of the world in parallel about 400,000 to 200,000 years ago [1,2]. Another striking period of human history is represented by the Neolithic Revolution with development from a foraging life-style (gathering and hunting) mostly associated with nomadic activities, to an agricultural activity of people settled in permanent areas of fertile land. Early activity of this type is described in particular along large rivers (Euphrates, Tigris and Nile; [3]). Introduction of fire and agriculture made it possible to feed more people and are prerequisites for development of the amazing expansion of the global population from about 5 million people 10,000 years ago up to 7 billion people today [4]. These few examples of nutrition illustrate the obvious fact that healthy and sufficient food is essential for population growth. 

### 1.1. Population Growth and Life Expectancy

Throughout most of human history, the pace of growth of the global population has been very slow [5]. World population reached 1 billion around 1800, and after another 125 years it was 2 billion. The world is currently in a period of faster population growth, increasing from 3 to 7 billion within the space of the past half-century [6]. In 2011, there was ~135 million births and 57 million deaths, a net increase of 78 million people [4]. According to the latest medium-fertility projections the world population will continue to grow throughout this century, reaching 9.3 billion in 2050 and 10.1 billion in 2100, obviously with a large degree of uncertainty. With the present trends there is a marked increase in age of most populations, both in the group of working age (15–65 years) and in the elderly (above the age of 60 years). 

During the last 150 years the life expectancy has increased by about 32 years in Norway for boys born in 2011 up to 79 years, while the life expectancy has increased by about 34 years for girls up to 83 years [7]. A similar trend is seen internationally with an increase during the last century of about 2.5 months per year. This striking increase in life expectancy in several countries is probably due to factors like improved quality of drinking water, more effective sewer systems, improved personal hygiene, effective vaccination programs and improved diet.

### 1.2. Modern Nutrition Research

In spite of the fact that life expectancy has increased markedly during the last few centuries all populations may benefit from optimized nutrition to reduce incidence of obesity, type 2 diabetes mellitus (T2D), cardiovascular diseases as well as several types of cancers and infectious diseases. Nutritional science should be focused on preventing development of diseases as well as supporting the repair processes important for curing already fully developed diseases [8]. Traditional nutrition research has contributed significantly to modern biomedicine and obviously promoted prolonged life expectancy. However, it is still a large potential for improving diet and health for many groups in economically developing as well as developed countries. This potential can be exploited by designing good studies and applying new and advanced techniques, mostly based on molecular methods and advanced biostatistics. 

### 1.3. Nutrigenomics and Molecular Nutrition

The National Institute of Health defines Genomics as the study of all of a person’s genes, including interactions of those genes with each other and with the person’s environment [9]. Genomics includes the scientific study of complex diseases such as T2D and cancer because these diseases are typically caused more by a combination of genetic and environmental factors (genetic interactions and gene-environment interactions) than by individual genes. Nutrigenomics is by definition a broader field of science than genomics. It is the study of the genome-wide influence of nutrition and the subsequent time-dependent response in transcriptomics, proteomics, and metabolomics to describe the phenotype of a biological system [10,11].

The concept of nutrigenomics has been introduced in particular in association with the establishment of the FP6 Network of Excellence named just Nutrigenomics Organisation [12], and with the publication of Müller & Kersten’s paper “Nutrigenomics: Goals and Perspectives” [10]. The concept of nutrigenomics has often been focused on the effects of nutrients and other food constituents on gene expression, in particular as ligands for transcription factors exemplified with the discovery of the nuclear receptors retinoid X receptors (RXRs) and peroxisome proliferator-activated receptors (PPARs) with retinoic acid and fatty acids (FAs) as ligands, where nutrients like FAs and derivatives of retinol can alter transcription of DNA to RNA. The influence of genetic variation on absorption, metabolism, elimination or biological effects of nutrients have also traditionally been included in the concept of nutrigenomics to optimize nutrition according to the subject’s genotype. 

**Figure 1:**
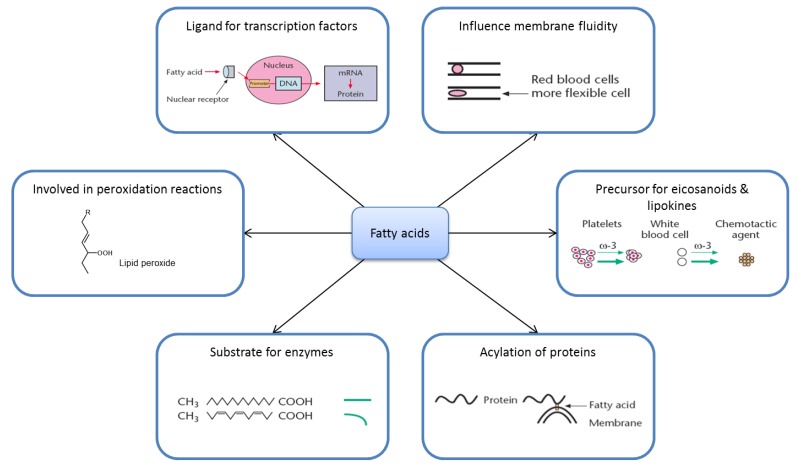
Molecular mechanisms of nutrients exemplified by fatty acids.

Although several great discoveries have been described with the nutrigenomic approaches, the understanding of how nutrients execute their biological effects also depends on mechanisms not acting through the genome (Figure 1). The concept of “molecular nutrition research” is broader than “nutrigenomics”, and may be defined as “Science concerned with the effect of nutrients and foods/food components on whole body physiology and health status at a molecular and cellular level”. The precise determination of molecular mechanisms underlying human health and disease offers a great potential for promoting health, and lowering mortality and morbidity, and includes the science of nutrigenomics.

In this review we will focus on old and in particular new technologies to describe the mechanisms of action by which nutrients execute their biological functions under different physiological conditions. It is a great potential for improving health by understanding the interaction between nutrients/foods and body functions, and thereby improve dietary prevention and treatment of diseases affecting people in affluent as well as poor societies.

## 2. Nutrition and Metabolism Are Complicated

### 2.1. Nutrition Is Demanding

In order to improve nutritional knowledge we have to consider the complexity of nutrition as outlined in Table 1.

**Table 1:** Nutrition is complicated due to many variables.

We eat ~1.5 kg food & drink ~2 L liquid/day; About 40 essential nutrients are known; Thousands of known compounds in foods without known biological functions; Thousands of unknown compounds in foods without known biological functions; About 10^13^ cells in the body & about 10^14^ bacteria in the GI tractus; Mostly unknown and complicated interplay between diet and the microbiome; Many organs & some hundreds of cell types are found in the body; About 25,000 genes in human cells; Human genome includes 3 billion base pairs; Some millions single nucleotide polymorphisms (SNPs); A large epigenetic variation between individuals due to environmental factors; About 100,000 transcripts (mRNA); About 100,000 proteins; About 1000 lipids & thousands of water-soluble metabolites.

Nutrition is complicated because of the multitudes of essential nutrients, known and unknown chemical compounds without known biological functions, different cell types and the extensive microbiological activity in the intestine, combined with a great genetic and epigenetic variation. All the variable factors allow an extensive variation between individuals as well as between different physiological states like fasted, fed, cold, warm, rested, exercised, exhausted, male and female, menstrual cycle, pregnant, lactating and age ranging from newborn to old. This extensive complexity of nutritional science demands advanced approaches to unravel the relations between diet and health for different ages, sexes and environmental conditions.

### 2.2. Fatty Acid Metabolism Is Complex

To illustrate the complexity of nutrition research we will describe some aspects of metabolism and mode of action of FAs (Figure 1).

An adult consumes approximately 85 g of triacylglycerol (TAG) daily. During digestion, free FAs (FFAs) and monoacylglycerols are released and absorbed in the small intestine. In the intestinal mucosa cells, FFAs are reesterified to TAG, incorporated in chylomicrons, transported to systemic circulation, and hydrolyzed to FFAs in capillaries mainly in muscle and adipose tissue.

**Figure 2:**
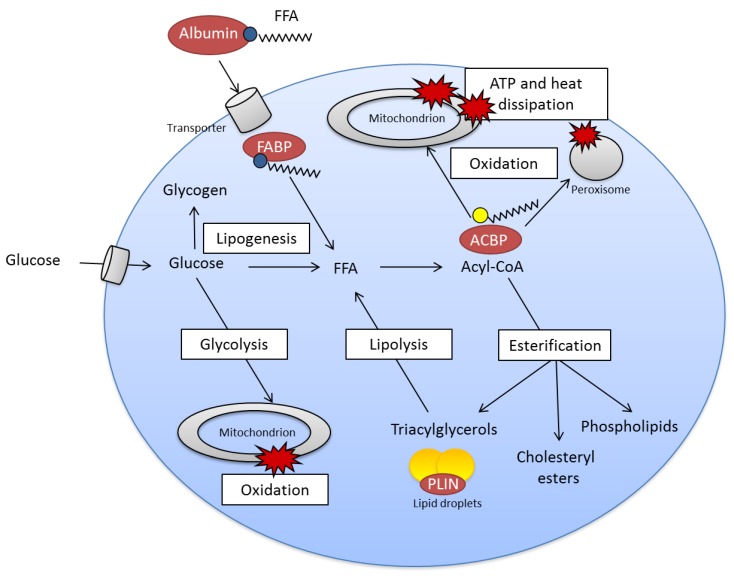
Simplified view of fatty acid metabolism.

FFAs enter cells mainly by FA transporters in the plasma membrane and are bound to FA-binding proteins (FABP) (Figure 2), activated to acyl-CoA before they are shuttled via acyl-CoA-binding protein (ACBP) to mitochondria or peroxisomes for oxidation to form ATP and heat, or to endoplasmic reticulum for esterification to different classes of lipids such as phospholipids, cholesteryl esters and TAG. FFA stored as TAG in lipid droplets may undergo lipolysis and reesterification. Glucose may be transformed to FFA (*de novo* lipogenesis) if there is a surplus of glucose/energy in the cells (Figure 2).

### 2.3. Ligands for Transcription Factors/Altered Gene Expression

FAs or their derivatives (acyl-CoA or eicosanoids) and phospholipids may interact with nuclear receptor proteins that bind to certain regulatory regions (promoter) of DNA and thereby alter transcription of these genes (Figure 1) [13]. The receptor protein may in combination with a FA function as a transcription factor. The first example described of this was PPAR. FAs as well as eicosanoids can bind directly to PPARα and PPARγ [14,15]. Strong activators of PPARα and PPARγ are unsaturated FAs such as oleic acids, linoleic acid (18:2, *n*-6), alpha-linolenic acid (18:3, *n*-3) and arachidonic acid (AA, 20:4, *n*-6). FAs may also influence expression of several glycolytic and lipogenic genes independent of PPAR. Polyunsaturated FAs (PUFA) may influence proliferation of white blood cells along with the cells tendency to die by programmed cell death (apoptosis) or necrosis. Thus, FAs may be important for regulation of gene transcription and thereby regulate metabolism, cell proliferation and cell death.

### 2.4. Eicosanoids

Eicosanoids are signal molecules formed from 20 carbon atoms PUFA derived from the diet. The most common precursor for eicosanoids is AA. These multitudes of signal molecules are called leukotrienes, prostaglandins, thromboxanes, prostacyclins, lipoxins and hydroxy-FAs. Eicosanoids are important for several cellular functions like platelet aggregability, cellular chemotaxis and cell growth. Eicosanoids are mostly synthesized in cells where they execute their effects, and they are rapidly formed and degraded. Different cell types produce various types of eicosanoids with different biological functions. For example, platelets mostly make thromboxanes, whereas endothelial cells mainly produce prostacyclins. Eicosanoids derived from very long-chain *n*-3 PUFA (mostly eicosapentaenoic acid (EPA, 20:5, *n*-3) are usually less potent than eicosanoids derived from *n*-6 PUFA [16].

### 2.5. Substrate Specificity

FAs may execute their action by having a different ability to interact with enzymes or receptors, as compared to other FAs. For example, EPA is a poorer substrate than all other examined FAs for esterification to cholesterol [17] and diacylglycerol [18]. Some *n*-3 PUFA are preferred substrates for certain desaturases [19]. The preferential incorporation of *n*-3 PUFA into some phospholipids, is caused by *n*-3 PUFA being preferred substrates for the enzymes responsible for phospholipid synthesis. 

### 2.6. Membrane Fluidity

When large amounts of *n*-3 PUFA are ingested, there is a high incorporation of these FA in membrane phospholipids, which may alter physical characteristics of the membranes. The very large amount of DHA in phosphatidylethanolamine and phosphatidylserine in certain areas of the retinal rod outer segments is probably crucial for the function of membrane phospholipids in light transduction, because these lipids are located close to the rhodopsin molecules. The flexibility of membranes from blood cells in animals fed fish oil, is markedly increased, and may be important for the microcirculation. Increased incorporation of very long-chain *n*-3 PUFA into plasma lipoproteins changes the physical properties of low-density lipoproteins (LDL) promoting reduced melting point of core cholesteryl esters [20]. 

### 2.7. Lipid Peroxidation

Lipid peroxidation products may act as biological signals. A major concern with intake of PUFA has been the high degree of unsaturation and thereby the possibility of promoting peroxidation of LDL. Modified LDL may be endocytosed by macrophages and initiate development of atherosclerosis. Oxidatively modified LDL has been found in atherosclerotic lesions, and LDL rich in oleic acid seems to be more resistant to oxidative modification than LDL enriched with *n*-6 PUFA in rabbits. It should be recalled that the dietary amount of saturated FAs, *trans*-FAs and cholesterol are the lipids that strongly correlate to development of coronary heart diseases, whereas the amount of dietary PUFA is related to reduced incidence of these diseases. Several studies suggest it is important to have the proper amount of antioxidants with the PUFA to decrease the risk of lipid peroxidation [21].

### 2.8. Acylation of Proteins

Some proteins are acylated with stearic (18:0), palmitic (16:0) or myristic acid (14:0) [22], thereby influencing anchoring or folding of certain proteins, which may be crucial for the function of these proteins. Although the saturated FAs are most commonly covalently linked to proteins, also PUFA may acylate proteins [23].

**Figure 3:**
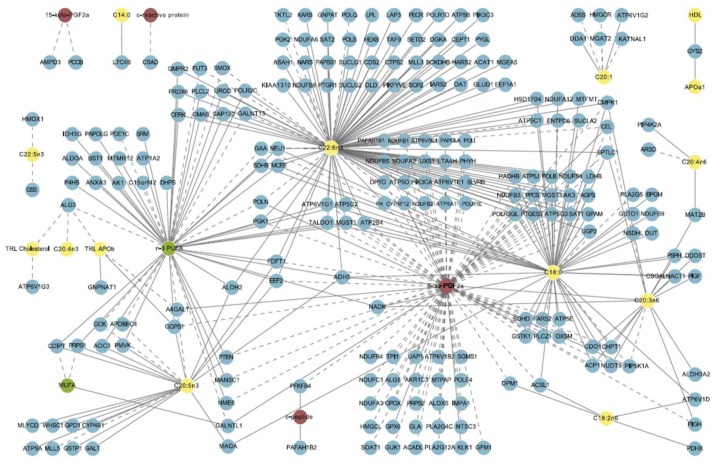
Network of associations between dietary intake, adipose gene expression, and phenotypic markers. Green nodes: nutrients; yellow: lipid, fatty acid, and apolipoprotein variables in blood; red: inflammatory and oxidative stress markers in blood; blue: gene expression (enzyme) in adipose tissue. Solid line: positive correlation/covariance; dashed line: negative correlation/covariance. Note: This figure is adapted with permission from [24], Copyright © 2011 Morine *et al*.

A few aspects of FA metabolism in cells have been focused in this section (Figure 2) but an extensive complexity of FA metabolism can be illustrated by the network of associations outlined in Figure 3. In this figure multivariate analysis identifies a strong relationship between dietary *n*-3 PUFA, adipose tissue gene expression and markers of metabolic health [24].

## 3. Methods in Modern Nutrition Research

Nutrients may influence gene expression “directly” as ligands for nuclear receptors or by inducing epigenetic modifications. However, nutrients are also essential building blocks (essential amino acids), may act as coenzymes in chemical reactions (vitamins), can be converted into bioactive products (fatty acids), inhibit oxidation of other molecules (antioxidants), or serve as energy sources. 

**Figure 4:**
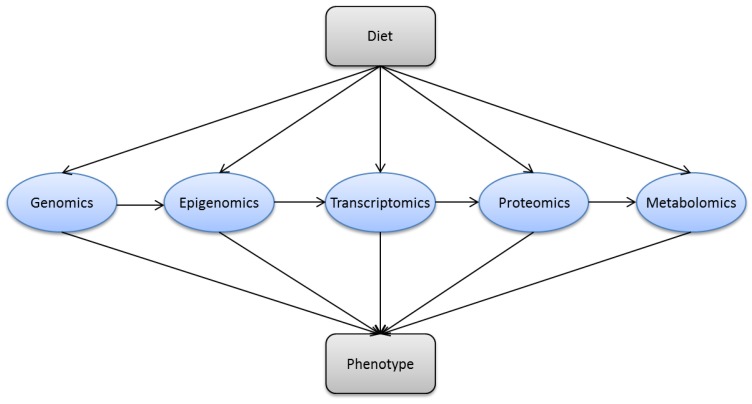
Dietary factors may interact with multiple biological processes. (Genomics) nutrients interact with genes and alter functional outcomes like dietary treatment of phenylketonuria; (epigenomics) nutrients may induce epigenetic changes like fatty acids promote methylation of the PGC-1α promoter; (transcriptomics) nutrients may influence gene expression as ligands for nuclear hormone receptors; (proteomics) nutrients may post-translationally modify proteins, e.g., protein-energy malnutrition leads to post-translational modifications of transthyretin; and (metabolomics) nutrients may change the metabolomic signature in the blood, e.g., carotenoids are biomarkers of fruit and vegetable intake.

New and advanced molecular techniques provide opportunities in nutritional science. These technologies are often based on the different *omics *(genomics, epigenomics, transcriptomics, proteomics and metabolomics) (Figure 4)*. *Some molecular methods, which can be applied in nutrition research (Table 2) will be put in context and explained in the sections below.

**Table 2:** Methods in nutritional research.

Research Area	Technologies	Assessed Parameters
Epidemiology	Observational	Association between diet and health outcomes and effects of controlled dietary changes
	Experimental	
Genomics	Microarray	Association between genetic variation (e.g., SNPs, alleles) and phenotypic traits
	Next generation sequencing	
Epigenomics	Bisulfite sequencing	DNA methylation and histone modification
	ChiP-sequencing	
Transcriptomics	Microarray	mRNA levels and splice variants
	RNA sequencing	
Proteomics	Chromatography	Protein composition and posttranslational modifications
	Electrophoresis	
	Mass spectrometry	
	Protein microarrays	
Metabolomics	Gas liquid chromatography	Metabolites
	Liquid chromatography	
	Mass spectrometry	
	Nuclear magnetic resonance	
Microbiota	Sequencing the 16S rRNA gene	Microbe species composition; genome, transcriptome, proteome and metabolome of the microbiotic community
	Metaomics (includes all omics described above)	
Imaging	CT	Whole body dynamic non-invasive detection of body composition (fat and lean mass), gene regulation and molecular tracers and probes
	MRI	
	PET	
	SPECT	
	Optical imaging	
Calorimetry	Indirect calorimetry	Energy intake and expenditure
	Direct calorimetry	
Cognition	Cognitive tests (K-ABC, Fagan, ERP, Kendrick object learning, Trail making, Digit symbol, Block design, Mini-mental state examination, Oral word association), EEG	IQ (sequential & simultaneous processing, nonverbal abilities, recognition memory)
Systems biology	Mathematical modeling	Integrate large data sets to understand complex physiological systems
	Statistical methods	

SNP: Single nucleotide polymorphism; ChIP: chromatin immunoprecipitation; CT: computed tomography; MRI: nuclear magnetic resonance imaging; PET: positron emission tomography; SPECT: single photon emission computed tomography; K-ABC: Kaufman assessment battery for children; ERP: evoked response potentials; EEG: electroencephalography.

### 3.1. Nutritional Epidemiology

Epidemiology is the study of determinants and occurrence of disease in human populations [25]. The main objective in nutritional epidemiology is to study the role of nutrition in causes and prevention of disease to ensure the highest quality of health recommendations [26,27]. Epidemiological research plays an important complementary role to experimental investigations in animals and *in vitro*, and can be used effectively to generate hypothesis for mechanistic studies [25,28]*.*

The key advantage of nutritional epidemiology is its direct relevance to human health, in contrast to findings from *in vitro* studies and animal experiments, which cannot be extrapolated directly to humans. However, the methods for collecting data on food intake are inaccurate and not easily reproducible [25,26]. Thousands of foods with different origin and composition are consumed in the modern societies. Although individual food habits are more limited than for the whole population, there is a big variation from day to day and seasonal, in addition to a significant change through the different stages of life [29,30]. No matter what methods are used for collection of dietary intake like 24 h recall, food frequency questionnaires or weighed food registration, we have serious challenges with under- and over-reporting or adjustments of food intake due to the data collection. Moreover, interpretation of data from epidemiological studies can be difficult because bias and confounding factors may affect the results [25,26]. Determining causality is impossible in observational epidemiological studies, whereas experimental studies give stronger evidence for causality. The disease process is often complex, and multiple risk factors may interact in development of disease. Observational epidemiology is primarily used to obtain disease information, measure prevalence and develop hypothesis about disease etiologies. Experimental epidemiology is focused on testing hypothesis and establishing the effect of dietary changes on health outcomes. 

**Figure 5:**
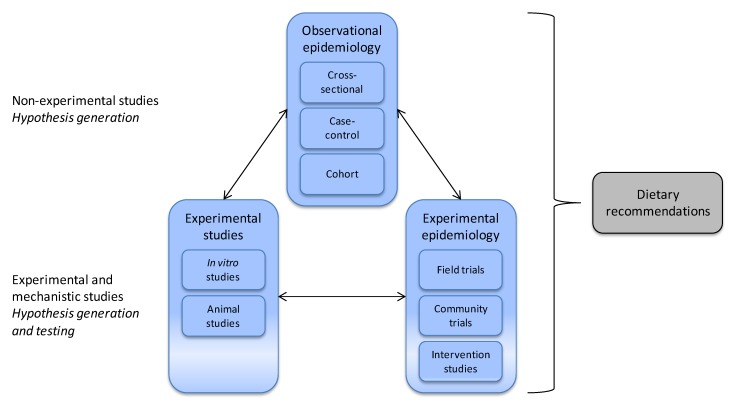
Epidemiological methods related to other studies in nutritional science. Observational epidemiology includes cohort, case-control and cross-sectional studies, whereas experimental epidemiology includes field trials, community trials and intervention studies. Observational studies help formulate hypothesis to be tested in subsequent experimental studies. Mechanistic studies are important for understanding physiological and biological mechanisms at cellular, tissue, and whole body level. When evidence is supported by a large number of data from *in vitro*, animal, and epidemiological studies dietary recommendations can be made.

Identification of a link between an exposure and health outcome often begins with an epidemiological study. An example is the association between obesity and elevated concentrations of several plasma amino acids. The type of dietary protein turns out to be associated with the risk of obesity, suggesting that specific amino acids may contribute to regulation of body weight [31,32,33]*. *Notably, in large epidemiologic studies, plasma total concentration of the sulfur-containing amino acid cysteine (tCys) is strongly and independently associated with fat mass and odds of obesity in adult populations [34,35]. Recently, the cysteine-fat mass relationship has been confirmed in younger subjects. Plasma tCys was associated with body fat percent and obesity in 984 children and adolescents (4–19 years) [36], and with waist circumference in 677 prepubertal children (6 to 11 years) [37]. However, because these findings are derived from non-experimental studies (Figure 5), interpretation must be performed carefully; high plasma concentration of tCys might promote obesity or obesity may influence cysteine metabolism and raise plasma tCys. Another possibility is that confounding factors may increase tCys and predispose for obesity, or that tCys might be a marker associated with obesity or obesity-related morbidity. 

To further clarify the molecular pathways and mechanisms linking cysteine and obesity, *in vitro* and animal studies have been carried out*. *Earlier *in vitro* studies have demonstrated that cysteine stimulates *de novo* lipogenesis and inhibits lipolysis [38,39]. Dietary cysteine supplementation decreases metabolic rate, induces lipogenic enzymes and increases adiposity in rodents, whereas dietary restriction of the precursor methionine has opposite effects [40,41]. This is consistent with human studies observing that vegetarian diets (low in methionine) are associated with low weight gain [32] and T2D risk [42]. Further evidence that cysteine is causally related to fat mass comes from studies in rodents as well as humans showing that genetic enzyme defects increasing or decreasing cysteine formation increase or reduce body weight, respectively [43,44]. Moreover, epidemiological and cell biological data suggest a redox mechanism, possibly via H_2_O_2 _signaling pathways [38,45], although this needs further investigation. Thus, cellular, animal and epidemiological data point to an obesogenic action of cysteine [46], although more research is required before we can conclude that cysteine is important for development of obesity.

Collective data from cellular, animal, and human studies are required to identify mechanisms, consequences and importance of potential links between the exposure and outcome, as illustrated by the possible involvement of cysteine in human obesity. No epidemiological study can alone provide an absolute answer about the effect of the exposure on the outcome [28]. When an association between a risk factor and the outcome is supported by evidence from a large number of observational studies, basic sciences about biological mechanisms, and experimental epidemiology, causality is strengthened and dietary guidelines may be provided [27] (Figure 5).

### 3.2. Genomics

The suffix “-ome” comes from the Greek for all, every, or complete. Genomics refer to the study of all the genes (the genome) of an individual, including interactions of those genes with each other and with the individual’s environment [9]. Strictly speaking, genomics does not include transcriptomics and epigenomics. Thus, in this section we focus on the interaction between diet and the genome and present separate sections on epigenomics and transcriptomics. 

The physiological effect of a nutrient depends on multiple processes such as digestion and absorption in the gastrointestinal tract, transport in the blood, uptake and metabolism in a variety of cells, and excretion via the kidneys and gastrointestinal tract. Each of these processes involves multiple gene products with polymorphisms, which potentially can alter the host’s physiological response to diet. 

Single nucleotide polymorphisms (SNPs) are defined as variations in DNA sequence where one of the four nucleotides is substituted for another [47]. SNPs may either have no consequence or a significant effect on the function of the gene product. Human genome-wide association studies (GWAS) take advantage of the inter-individual differences due to genetic polymorphisms by examining the statistical association between millions of SNPs in a large population and the phenotypic trait of interest [48]. Recent GWAS have provided many loci implicated in the development of chronic diseases such as T2D [49]. However, these loci explain only a small fraction of the total genetic component. For example, obesity has an estimated heritability of about 65%, whereas large human GWAS explain less than 3% of the genetic component [50]. 

Identifying the relevant genes contributing to complex diseases is difficult, because several genes with small effects contribute to overall heritability. Moreover, GWAS alone does not have sufficient power to demonstrate interactions among genes or between genes and the environment, and it is difficult to move from locus to disease pathway directly in humans [51]. To simplify genetic analysis, natural variations relevant to disease have been studied in rodents. This has traditionally involved linkage-mapping methods with crosses between strains to identify quantitative trait loci (QTLs). The advantage of performing GWAS in inbred strains of mice is that we can control environmental exposure, select strains with large phenotypic variations, and map genes for complex traits with high resolution compared to linkage mapping [52]. An association-based approach called the hybrid mouse diversity panel, using more than hundred classical inbred strains, has the potential to identify gene-environment interactions and to map genes with a resolution of less than a megabase [53]. 

Recent follow-up studies to large human GWAS have been designed to show gene-diet interactions. The studies were focused on if specific dietary compounds might modulate the phenotypic effect of a certain genetic variant. A classical example of gene-diet interactions is the dietary treatment of phenylketonuria (PKU). PKU is a genetic disorder caused by a mutation in the gene encoding the hepatic enzyme phenylalanine hydroxylase. This enzyme is required to metabolize phenylalanine into tyrosine. In PKU the enzyme is absent, and too much phenylalanine accumulates in the body causing mental retardation. However, when newborns are diagnosed with PKU they can get a phenylalanine-free diet, which prevents the neurotoxic effects of high blood levels of phenylalanine [54]. The FTO (fat mass and obesity associated) gene is a good example on how variation in gene sequence interacts with environmental factors to determine phenotype, because carriers of one or more “risk” alleles have a 1.5 kg higher body weight per allele [55,56]. Although the absolute effect size is modest, it should not be underestimated at a population level, as the association potentially may influence body weight of up to half of the world’s population [57]. Observational studies suggest that high dietary saturated fat intake accentuate the susceptibility for obesity in carriers of the FTO risk allele [58,59]. Furthermore, a recent meta-analysis showed a 27% attenuation of the association between FTO risk allele and the degree of obesity in physically active adults, highlighting the importance of physical activity at least in some individuals predisposed to obesity [60].

Thus, it is important to know how different genotypes may interact with environmental factors to understand the effect of genetic polymorphisms on health.

### 3.3. Epigenomics

One of the first assumptions of adverse environmental impact on normal fetal development was described by Barker and Osmond [61], often referred to as Barker hypothesis. By using epidemiological data they linked low birth weight with increased risk of death from cardiovascular disease [61]. Several other studies have made similar observations associating early adverse conditions with metabolic dysfunction later in life; the Dutch famine cohort showed increased risk of cardiovascular diseases 4–5 decades later among children born to mothers who experienced extremely severe undernutrition during the first trimester of pregnancy [62]. These interesting epidemiological data in addition to major advances in the field of genetics led to a new research field investigating the connection between epigenetic modifications and environmental effects such as dietary intake. Epigenetic refers to modifications impacting gene expression occurring without changes in nuclear DNA base sequence [63]. Epigenomics can be defined as the study of the complete set of epigenetic modifications in a cell or a tissue at a given time.

The human body consists of more than 200 different cell types with the same DNA sequence but unique gene expression patterns. The difference in gene expression between the cells is mainly governed by epigenetic modifications, including changes in DNA methylation and histone modification. DNA methylation is one of the major epigenetic modulators [64]; it can suppress gene expression by modulating the access of the transcript machinery to the chromatin or by recruiting methyl-binding proteins [65]. Because DNA methylation is mitotically stable, the assumption has been that environmental factors were unlikely to induce significant changes in DNA methylation pattern in normal adult tissues. However, recent studies support the notion that environmental factors affect metabolic functions through epigenetic modifications. 

Twin studies have shown that DNA methylation profiles were more divergent in older twins than in infant twin pairs, suggesting that environmental factors may influence the epigenome [66]. Diet-induced weight loss for 8 weeks in obese men altered DNA methylation in peripheral blood mononuclear cells of specific genes [67]. Changes in DNA-methylation levels among humans with metabolic diseases were associated with alterations in expression of genes involved in mitochondrial function, including PGC-1α [68]. Reduced PGC-1α activity is linked with the pathogenesis of metabolic diseases as it increases metabolic and cardiovascular risk and precedes the development of T2D [69,70]. Interestingly, whereas palmitate and oleate can acutely induce methylation of the PGC-1α promoter, exercise induces hypomethylation of PGC-1α in skeletal muscle [71,72]. The hypomethylation of the PGC-1α promoter in response to exercise was paralleled with an increase in PGC-1α mRNA content [72]. 

Transgenerational epigenetic inheritance refers to phenotypes present in successive generations caused by epigenetic modifications passed via the gametes [73]. Until recently, epigenetic modifications have been considered erased during gametogenesis or early embryogenesis. However, novel findings have shown that epigenetic marks are not always cleared between generations [74]. A well-studied model of transgenerational epigenetic inheritance is the Agouti mice [63]. The groundbreaking study of Wolff and colleagues showed that methyl-supplementation of the maternal diet induced epigenetic regulation and altered the Agouti gene expression in the offspring causing altered fur color [75]. A striking example of transgenerational effect on metabolic disease was published by Ng *et al.* [76]. They showed that when male rats were fed a high-fat diet before mating their female offspring inherited programmed β-cell dysfunction. This phenotype was associated with variation in the methylation pattern of the *Il13ra2* gene. In humans, the epigenetic state at birth may predict later childhood adiposity [77]. In two independent cohorts greater methylation of RXRA at birth was strongly correlated with larger adiposity in later childhood. Furthermore, early carbohydrate intake during pregnancy was inversely associated with childhood adiposity [77].

The study of microRNAs is often classified to be part of epigenetics. MicroRNAs are small non-coding RNA molecules derived from hairpin precursors, usually between 20 and 30 nucleotides in length [78]. They can mediate post-transcriptional silencing for about 30% of protein-encoding genes in mammals by pairing with complementary sites in the 3′ untranslated regions of target genes. Interestingly, a recent study showed that exogenous plant food microRNAs can regulate target genes in mammals [79]. Zhang *et al.* [80] showed that MIR168a, a plant microRNA, may pass through the gastrointestinal epithelium and enter the blood and organs. Furthermore, they showed by *in vitro* and *in vivo* studies that MIR168a can bind to the human/mouse LDL receptor adaptor protein 1 (LDLRAP1) mRNA, inhibit hepatic LDLRAP1 expression, and reduce LDL removal from plasma.

In conclusion, several lines of evidence indicate that some of the effects of diet and physical activity are induced via epigenetic modifications.

### 3.4. Transcriptomics

Transcriptomics refers to the complete collection of gene transcripts in a cell or a tissue at a given time [10], and may be used to study gene transcription in response to dietary changes [81]. The nuclear hormone receptor superfamily of transcription factors is probably the most important group of nutrient sensors, which influence gene expression. Numerous nuclear hormone receptors, such as RXR, PPARs, and liver X receptor (LXR), bind nutrients and undergo a conformational change that results in the coordinated dissociation of co-repressors and the recruitment of co-activator proteins to enable transcription activation.

RNA microarray technologies and sequencing can be used to evaluate the interactions between diet and genes measured as changes in genetic expression. When applied together with traditional biochemical methods, transcriptomics provide more extensive information about nutrition status and metabolic responses to diet. Transcriptomics is mainly used for three different purposes in nutrition research (reviewed in [10]); first, it can provide information about the mechanism underlying the effects of a certain nutrient or diet; second, transcriptomics can help to identify genes, proteins or metabolites that are altered in the pre-disease state and might act as molecular biomarkers; third, transcriptomics can help to identify and characterize pathways regulated by nutrients.

Human dietary intervention studies have successfully used transcriptomics to show that diet induces alterations in gene expression [79,82]. However, an important challenge in human transcriptomics studies is the inaccessibility of human tissues. Blood, subcutaneous adipose tissue, and skeletal muscle are among the tissues, which can be relatively easily collected. Thus, animal studies can be good supplements to human studies to understand how nutrients affect gene regulation in a variety of tissues. A good example on how transcriptomics can be used is the study of Caesar and colleagues [83]. The authors set out to study the effects of expanding mesenteric adipose tissue in a murine model. They performed microarray analysis on mesenteric, subcutaneous, and epididymal adipose tissues after up to 12 weeks of high fat feeding. Interestingly, they discovered that high fat feeding induced similar reduction in subcutaneous and mesenteric adipose tissue *de novo* lipogenesis, whereas the gene expression in epididymal adipose tissue was unaffected. Follow-up analysis with targeted lipidomics and biochemical analysis showed that *de novo *lipogenesis was down-regulated in the distal epididymal adipose tissue and that this specialized adipose tissue might promote elongation and desaturation of some essential PUFA for spermatogenesis.

### 3.5. Proteomics

Proteomics represents the large-scale study of the entire set of proteins expressed in a given cell, tissue, or organism at a defined time-point. Most biological functions are transmitted via proteins like enzymes, receptors and structural components. Studying proteins directly is necessary because gene expression levels do not always correspond to protein abundance because protein levels are determined by regulatory input from synthesis to degradation. Secondly, pre-mRNA transcripts might give rise to several proteins because of alternative splicing. Thirdly, subcellular localization is important for biological effects. In addition, posttranslational modifications and interactions with other proteins or RNA affect protein action and activity. Diet can induce post-translational modifications of proteins. One example is the study of Henze and colleagues showing that protein-energy malnutrition leads both to changes in transthyretin concentration in the blood and post-translational modifications of the protein [84].

**Figure 6:**
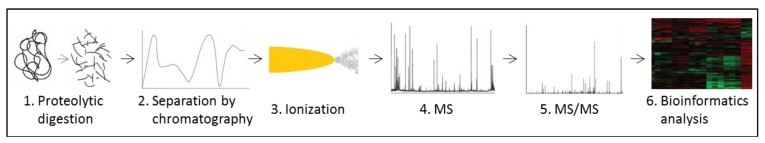
A common workflow in a proteomic experiment. Protein samples can be derived from tissues, plasma, cultured cells or organelle fractions. Proteins are digested (**1**) and the resulting peptides are separated by chromatography (**2**), ionized (**3**) and the mass-to-charge ratio (*m/z*) is measured in an initial scan (**4**). To identify the amino acid sequence, peptides are selected for fragmentation and subjected to MS/MS (**5**). Finally, bioinformatics tools are used to identify and/or quantify the proteins in the sample (**6**).

Several review articles on the use of proteomics in nutrition research have been published [85,86,87]. The focus has been on identifying new health biomarkers and bioactive peptides in foods. Among the potential approaches for studying the proteome in large scale, chromatography combined with mass spectrometry (MS) has become a leading method (Figure 6). Other techniques include one- and two-dimensional gel electrophoresis and antibody-based assays such as protein microarrays [88].

Current MS technology makes it possible to analyze several thousand proteins in a single sample, and is used for identification and quantification of proteins, as well as characterization of post-translational modifications and protein interactions [89,90]. Several approaches are used, but the method often involves digesting proteins in the sample into peptides (e.g., with trypsin) and fractionating the peptides (often with liquid chromatography (LC)) before subjecting the sample to MS analysis. Peptides are ionized and the mass-to-charge ratio measured. Most often two or several mass analyzers are used in sequence separated by a fragmentation step. This is called tandem MS, or MS/MS. The generated spectra can be used to determine the amino acid sequence, and thereby identify the proteins by bioinformatics tools. This approach was used in a study investigating the skeletal muscle secretome [91]. Skeletal muscle secretes peptides in response to muscle contraction that exert either paracrine or endocrine effects. These peptides are termed myokines, and might be involved in mediating the beneficial effects of physical activity on health. Proteins secreted by cultured human myotubes were identified by LC-MS analysis of the conditioned cell culture medium. Two hundreds and thirty-six proteins were detected, and 15 of the secreted proteins had enhanced mRNA expression in biopsies from *m. vastus lateralis* and/or *m. trapezius* of healthy individuals after 11 weeks of strength training.

MS can also be used for relative or absolute quantification of peptides, and the different methods either apply labeling of peptides before MS analysis or use label-free approaches based on spectral features [90]. Isobaric Tags for Relative and Absolute Quantitation (iTRAQ) are stable isotope containing tags that covalently bind all peptides in a sample [92]. Stable isotope labeling with amino acids in cell culture (SILAC) is a metabolic labeling technique where cells (and their proteomes) are labeled by growing them in medium containing heavy or light isotopes of essential amino acids [93]. Heavy isotope-labeled model organisms (such as rodents) are also available, allowing *in vivo* studies [94]. These labeling techniques have in common that differentially labeled samples are pooled and peptides sequenced and quantified simultaneously in one run. 

Forner *et al.* used SILAC to compare the mitochondrial proteomes of white and brown adipose tissue in mice [95]. This was achieved by comparing each tissue to a SILAC labeled control fraction from cultured cells. Several interesting differences were found [95]. Hwang *et al.* used a label-free method to analyze changes in protein abundance in skeletal muscle in relation to insulin resistance, and found a reduced abundance of mitochondrial proteins (among others) as compared to muscle tissue of healthy subjects [96]. To determine changes in lysine acetylation of mitochondrial proteins during energy restriction, a study on mice used a label-free approach and found dramatic, tissue-specific alterations [97]. Acetylation of mitochondrial proteins was primarily regulated in brown adipose tissue and liver. In liver, MS was used to identify specific proteins with altered acetylation, and 72 candidate proteins involved in metabolic pathways were found.

Selected reaction monitoring (SRM) is a targeted mass spectrometry technique that is emerging in the field of metabolomics as well as proteomics as a complement to untargeted shotgun methods [98]. This method is particularly useful when predetermined sets of proteins, such as those constituting cellular networks or sets of candidate biomarkers, need to be measured across multiple samples in a consistent, reproducible and quantitatively precise manner.

In recent years there has been a striking technological progress in the field of proteomics. Application of tryptic digestion, chromatography, MS, antibodies and bioinformatics in combination with other biochemical techniques, opens many new opportunities in future nutritional research.

### 3.6. Metabolomics

Metabolomics refers to the types and concentrations of all metabolites in a biological sample. Biological metabolites are specific products of genomic, transcriptomic and proteomic processes of the host or external organisms, as well as intrinsic and extrinsic influence on these. The characteristics and concentrations of all small molecules, water- as well as lipid-soluble, provide a potential for measuring flux through all important biological pathways, and thereby allow detailed understanding of how metabolites interact with tissue components of functional importance [99]. Metabolomics can also be used to identify biomarkers for intake of specific nutrients and health. For example it has recently been shown in an meta-analysis that blood concentrations of carotenoids, a biomarkers for fruit and vegetable intake, are more strongly associated with reduced breast cancer risk than are carotenoids assessed by dietary questionnaires [100]. 

Ideally, metabolomics should have the ability to provide a detailed snapshot of biological processes at any particular point in time. In nutritional research, such an approach may provide an opportunity to identify changes in metabolic pathways induced by nutrients or other life-style factors, to explore relationships between environmental factors, health and disease, and to discover novel biomarkers [101,102]. However, due to the diverse chemical nature of low-molecular metabolites, including lipids, amino acids, peptides, nucleic acids, organic acids, vitamins, thiols and carbohydrates, the global, untargeted analysis represent a tough challenge. Although development of analytical platforms enables separation, detection, characterization and quantification of a large number of metabolites from only minor amounts of biological samples [103], targeted metabolomics are most often used [104]. 

Targeted analysis, where a pre-defined set of metabolites are monitored, may be used for assessment of single nutrients or metabolites [105], determination of subsets of metabolites, including lipids [106], inflammatory markers [107,108] or oxidative damage [109,110]. The profiling of lipids has developed into its own field of lipidomics, and as adversely altered lipid metabolism is an underlying factor in a number of human chronic diseases, lipidomics has become an important tool to identify potential novel therapeutic targets [111,112]. 

Although metabolomics gain increased interest in nutrition research, there are still some major limiting factors. In untargeted metabolomics, there are many unidentified metabolites. The high number of “unknown” signals makes it often difficult to extract meaningful information. Thus, there is a great need for publically available databases for the identification of metabolites [101]. Furthermore, the use of pattern-recognition techniques is crucial for exploring novel molecules that may serve as biomarkers. Moreover, the data sets based on metabolomics are usually huge and multi-dimensional. The metabolomics data should be compiled along with data on transcriptomics and proteomics, supporting more extensive use of bioinformatics including multivariate analyses [113].

Due to the recognized and huge intra-individual variation, there is a need for standardization of study design, use of large study cohorts and homogenous study populations based on preliminary phenotyping of study subjects.

### 3.7. Microbiota

The human gastrointestinal tract is estimated to host up to 10^14^ microorganisms, tenfold the number of human cells, predominately composed of bacteria but also archaea, protozoa and fungi. Together they make up the gut microbiota, which during normal circumstances live in a commensal or mutualistic relationship with their host. Their central functions in immune defense and nutrition have led investigators to designate the gut microbiota as an organ by itself [114,115,116,117]. Although humans can live with a bacteria-free intestine the microbes are crucial for human health. For example, the gut microbiota metabolizes indigestible carbohydrates to valuable short-chain FAs; synthesize certain vitamins; degrade oxalates and is essential in recirculation of bile acids. 

Traditional *in vitro* cultivation has limited the research on gastrointestinal bacteria because their normal growth environment is complex and difficult to imitate. Thus, the introduction of gene-sequencing of the hypervariable region in the bacterial gene for 16S rRNA on amplicons from faecal samples has markedly extended the knowledge about their species diversity [118,119,120]. Between 500 and 1000 different species seem to occupy a single human gut, whereas the total microbiome in humans include between 10,000 and 40,000 species. However, the majority of microbes within the digestive tract appear to include less than 100 different species [121,122]. 

Development of next-generation sequencing have permitted mapping of the microbial metagenome in humans [123]. As part of the Metagenomics of the Human Intestinal Tract project, faecal specimens from 124 European individuals were analyzed and an average of 4.5 Gb (ranging between 2 and 7.3 Gb) of sequence was generated from each sample [124]. Genome annotation provided a set of 3.3 million non-redundant microbial genes and detected some 536,000 prevalent unique genes in each individual. Furthermore, almost 40% of the genes from each individual are shared with at least half of the individuals in the cohort. Among these are genes involved in the biosynthesis of short-chain FAs, amino acids and certain vitamins, which all are molecules suggested to be provided by bacteria to humans. 

How nutritional habits interfere with the intestinal microbiota is far from understood. It was traditionally believed that the microbe composition was relatively unchangeable, but DNA sequence analyses have challenged this view. Studies have clearly shown that the composition of gut microbiota adapt during changes from breast milk to solid food and when altering the composition of ingested macromolecules [125]. A recent evaluation of long-term dietary habits and short-term interventions concluded that small shift in microbe composition is prevalent after only one day, but that a period of a year have significantly more influence on the gut microbiota [126]. Changes in intake of non-digestible carbohydrates can affect faecal microbiota composition. A three-week controlled dietary intervention in obese males showed especially that resistant starch influenced the abundance of several dominant phylotypes [127]. Moreover, supplementation with galacto-oligosaccharides or inulin cause increased content of bifidobacteria in the gut [128,129]. Notably, non-responders are often observed and the outcomes appear to be more dependent on the initial composition of the individual gut microbiota than the dietary interventions [130]. 

Whereas, metagenomics provides insight into the genetic potential of microbiota, additional transcriptomics, proteomics and metabolomics analyses in combination with interventions and monitoring of the host, are necessary to develop biological model systems of functional significance for the gastrointestinal microbiota.

### 3.8. Imaging

Imaging in biomedicine represents a broad scientific and clinical approach usually distinguished based on spatial resolution (microscopic to macroscopic) or types of energy detected, like ionizing radiation, photons or sound waves [131]. Traditionally, imaging has been used to characterize morphological and anatomical properties, but during the last decade a new and important discipline called “molecular imaging” has emerged. European Society for Molecular Imaging defines molecular imaging as the characterization of the dynamics of molecular processes in living organisms. In comparison to “omics” approaches that provides comprehensive snapshots of biological indicators or biomarkers, molecular imaging advances this information, showing non-invasively the activity of markers and changes in location with time. The modalities in molecular imaging are positron emission tomography (PET) and single photon emission computed tomography (SPECT) that detect β- and γ-radiation; nuclear magnetic resonance imaging (MRI) that detect differences in relaxation time; photo-acoustic imaging that detect ultrasonic waves; and optical imaging that mainly record luminescent and fluorescent light.

Body composition is highly relevant in nutritional science and both computed tomography (CT) and MRI can distinguish between different tissue types and thus be used to reconstruct major anatomical compartments and tissues *in vivo*, thus providing direct quantification of either cross-sectional area or volume [132]. For example, MRI has been carefully validated with dissection of adipose depots in rats fed different types of fatty acids [133,134], and the data are in accordance with each other. The quality of the CT analysis is well established, but due to the damaging radiation risk of CT the use of MRI is expanding. The two methods have in comparative studies shown somewhat different results, but the use of higher magnetic field and improved MRI imaging procedures have reduced this discrepancy [135,136]. A potential confounding factor when attempting to quantify e.g., muscle size is infiltration of fat. However, post hoc analytical techniques that separately quantify contractile and non-contractile compartments within muscle tissue have been developed [137]. 

Essentially there are two approaches used in molecular imaging: (1) administration of molecular probes, which recognize and bind to a particular biological molecule or are activated by a specific process (e.g., enzymatic reaction); (2) reporter genes that are expressed in response to a gene regulatory event.

The imaging technologies PET and SPECT are based on detection of radioisotopes, which can be used to label a broad range of biological molecules. The most obvious differences between these two methods is that PET imaging exhibit the highest sensitivity, while the longer half-life of SPECT emitters offers a wider observational time-window. In nutritional science the most used radio-label probe is (18-F)fluro-D-glucose (FDG), which among others is used to explore glucose intolerance. In optical imaging, fluorescent molecules or chemiluminescent reactions are detected. This involves substantial structural change of labeled molecules, consequently changing the biological properties. However, genes encoding such molecules can stably be integrated in the genome offering the possibility of engineering reporter constructs as in transgenic reporter mice [138]. This enables timely unlimited studies of dietary effects on gene regulation. For example, transgenic mice reporting NF-κB activity have been used to show anti-inflammatory effects of dietary plant extracts [139].

### 3.9. Assessments of Energy Expenditure Using Calorimetry

Modern society has developed sophisticated methods to improve production, storage and distribution of nutrients. Thus, malnutrition occurs rarely in developed countries, but we suffer from an excess of energy supply. Tight control of energy intake and expenditure is essential for survival and a healthy life. Organisms have evolved to promote anabolism to store energy when energy supply exceeds demands and switch to catabolism when supply is lower than demand. Whereas some people have a remarkably stable weight over time, others steadily gain weight through a constant net surplus intake of energy. An important aspect of modern nutritional science is to identify genetic, nutrient and environmental factors promoting a stable body weight.

Identification of leptin as a genetic determinant of the obese phenotype model (ob/ob) [140] awakened interest in the analysis of energy expenditure in mice. Our ability to alter the genome in mice, either by deleting genes using knock out approaches, or by over-expressing genes, often results in mice with changes in body weight or body composition. Mouse genetics has therefore a great potential to increase our understanding of energy metabolism. Sophisticated methods to evaluate if food intake or energy expenditure (or both) contribute to the altered phenotype, may be used to analyze genetically modified animals.

**Figure 7:**
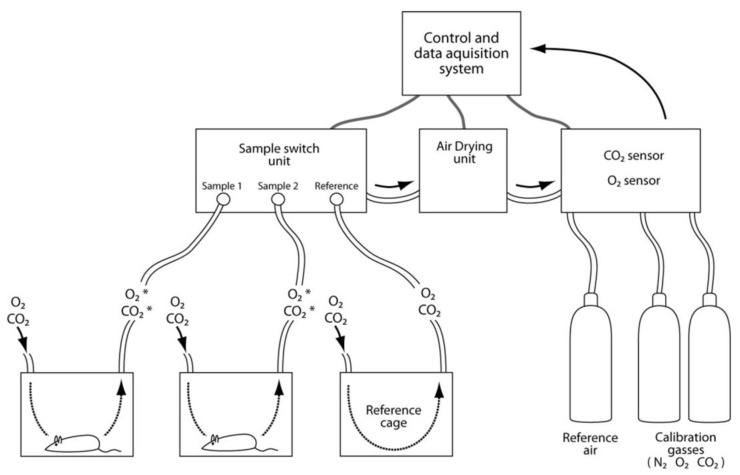
Indirect calorimetry. The typical indirect calorimetric system includes several gas-tight chambers (here illustrated by two housing cages and a reference cage), control units, control system and gas bottles. During the experiment, air of similar origin is delivered simultaneously to all chambers via the sample switch unit. Gas from each container is sequentially sent to the air-drying unit prior to CO_2_ and O_2_ measurements. The reduction in O_2_ and production of CO_2_ in chambers housing mice is calculated against the measured values of these gasses coming from the reference cage. Modern O_2_ and CO_2_ sensors have fast response times of minutes, which enable measurements of 10–20 cages several times per hour. The calorimetric chambers can be combined with devices for measurements like food and liquid intake, body weight, movement, voluntary exercise (e.g., running wheels), temperature in the cage, body temperature, and collection of feces and urine.

Energy expenditure can non-invasively be determined by direct or indirect calorimetry. Direct calorimetry assesses energy expenditure by direct measurement of heat produced by the animal [141] (Figure 7). The animal is placed inside an insulated chamber and the produced heat is measured using a calorimeter. Calorimeters are expensive with slow response time and they do not provide information about the nature of the oxidized substrates. Although direct calorimetry is the only method that can accurately quantify heat production and metabolic rate (MR) in metabolically normal as well as abnormal states, the technique is rarely used. With indirect calorimetry, energy expenditure is calculated based on accurate measurement of O_2_ consumption and CO_2_ production. This type of calorimetry has been applied in clinical settings and can be done in several ways [142]. A typical indirect calorimetric system includes a set of gas-tight chambers, which are ventilated with a steady flow of fresh air. Once an animal is placed inside a chamber, it will consume O_2_ and produce CO_2_. The decrease in O_2_ and increase in CO_2_ in each chamber is calculated against a reference chamber. The fast response time for the O_2_ and CO_2_ sensors enable sequential measurement of the chambers at 1–3 min intervals. In a typical experiment, 10 cages can be measured 4 times per hour. In addition to the speed, measurement of changes in the two gasses enables assessments of respiratory exchange ratio (RER), which is defined by the ratio of CO_2_ exhaled and O_2_ inhaled. Metabolism of carbohydrates gives a theoretical RER of 1.00, for protein the value is 0.83, and 0.70 for fat. Identification of the primary substrate oxidized gives additional information about the phenotype. Due to its simple measurements, indirect calorimetry has become the gold standard for assessment of energy expenditure. However, it is important to be aware that indirect calorimetry relies on assumptions that have never been tested to be accurate for genetic or pharmacologically induced changes in metabolic fuel partitioning affecting storage or promoting obesity and T2D [141].

Although equipment to measure energy expenditure is available, it is far from trivial to perform experiments giving reliable data. Indeed, there is controversy on how to analyze and interpret energy expenditure data obtained by indirect calorimetry as recently discussed in a guide to perform energy expenditure experiments [143]. A major challenge with energy expenditure studies is to evaluate if food intake or energy expenditure (or both) contribute to the altered phenotype. A consistent small alteration in energy intake or energy balance over a longer period of time may have significant effects on body weight and body composition. Because measurements typically are performed during a short time, identification of such small changes requires impractically large sample sizes to get enough statistical power. In addition, the phenotypic changes themselves pose problems. Once obesity or leanness has developed, behavioral and metabolic alterations that originally triggered changes in body weight may obscure or confound the processes that caused the phenotypic changes. Analysis of two different groups of mice, with different weight or body composition poses problems with normalization because various organs differ in metabolic rate. White adipose tissue (WAT), which is the tissue that expands with obesity, is metabolically less active than brain or liver. However, transformation of WAT to brown adipose tissue (BAT) enhances metabolic activity to become one of the most active tissues in the body. 

Despite the listed problems, energy expenditure measurements are widely used to evaluate changes in energy metabolism. Energy expenditure measurements are often combined with non-invasive body composition analysis (MRI or CT) to determine lean tissue mass (total body weight subtracted by fat mass). Three methods have been routinely used to normalize energy expenditure data. None of these are reliable if there are substantial differences in body weight or body composition among the individuals tested [144]. When evaluating earlier studies, it is important to understand the biases caused by the different normalization methods: (1) Normalization against total body weight. Increasing body weight is mainly caused by accumulation of adipose tissue with a low metabolic rate. Hence, this type of normalization has a tendency to give a false reduction in energy expenditure with increasing body weight; (2) Normalization against lean body weight. This way of normalization gives a false increase in energy expenditure. Although adipose tissue has a low metabolic rate, accumulation of WAT increases total body energy demand; (3) Normalization against “metabolic body” weight. The relationship between mammalian basic metabolic rate and body weight is proportional to body weight to the power of 0.75 [143]. Although this scaling seems correct when comparing different species, it is not accurate for animals within the same species with an imbalance in organ weights and altered body composition. To overcome problems with normalization, it has been suggested to analyze and plot energy expenditure for individuals and evaluate differences in energy expenditure among groups with ANCOVA [143].

Due to the difficulties with assessment of energy expenditure, it is vital to link a possible difference in energy expenditure to other types of observations. Usually, changes in energy expenditure can be explained by alterations in food intake, absorption of nutrients, physical activity, shivering, or RER. Altered thermogenesis can be involved in circumstances with altered UCP1/BAT uncoupling.

### 3.10. Systems Biology

Systems biology is a fashion-word in modern biology used to describe all aspects of a biological system in an integrated view. The fundamental principle is that the perspective on the whole organism will provide a more accurate view than the sum of the parts, based on the idea that a complex system has intrinsic properties that cannot be derived directly from the additive effects of its individual parts [51]. As of today, the most advanced approach of system biology is to integrate the information obtained from advanced technologies to describe and predict how the whole organism will react to certain environmental or genetic alterations. Typically, systems biology includes the information obtained from individual studies on genetics, epigenomics, transcriptomics, proteomics, metabolomics and functional assays including imaging, assessment of energy expenditure or the use of challenging test (OGTT, physical activity, intervention with different diets or meals, fasting). By extracting biological knowledge from a variety of technologies, integrative systems biology may provide predictive models of cells, organs, complex biochemical processes as well as entire organisms. Such integrative information may be used for the purpose of identifying new molecular targets of dietary exposure as well as biomarkers of disease [145].

System-based nutrition studies typically include five steps (reviewed in [51]). The first step would be to define the “system” to investigate. A system could be a cell population, an organ, or an entire organism, like experimental animals or humans. The second step would be to decide which information (components) that should be obtained from the system. In a transcriptomics study the authors would typically include gene expression data. The third step would be to determine how the components interact with each other. For example, co-variation between genes can be investigated. Fourthly, the investigators should model the dynamics of the system to understand the interactions between its components. For example, time-dependent gene-interactions changes can be studied in response to a high fat diet. Lastly, the model should be validated using experimental perturbations. In a molecular nutrition study you can for example use *in vitro* cell systems or genetically modified mice to validate the findings. 

An important asset in advanced systems biology is the possibility to compare experimental data extracted from diverse available databases. Ng *et al.* published in 2006 a collection of 150 publicly available databases [146]. Since then, the amount of new available data has expanded rapidly. One example on such a database is the nutritional phenotype database (dbNP), initiated by NuGO [147]. Following from this, the heterogeneity of experimental data, within and between populations, represent one of several challenges for systems biology, and future efforts should aim at the inclusion of full descriptions of experimental conditions upon the entering of data in public databases. 

Advanced simulation tools are of great importance to process and interpret the massive amount of data obtained in systems biology. There are numerous efforts directed at developing a human physiome, an extensive integrative model of human physiology that can be used for hypothesis testing as well as education [148]. The review by Ng *et al.* also includes an extensive list of available tools and resources, which may be of relevance to systems biology [143].

Systems biology can be a powerful tool in nutritional research to develop targeted nutritional strategies. However, when extrapolating results from specific studies for the purpose of understanding the whole organism, it is of great importance to consider the organism as a highly complex system comprising multiple feedback mechanisms to a number of inputs including dietary intake. For example, although linear responses may be observed in experimental models, the response of the human organism to extrinsic challenges is rarely linear and the output of individual phenotypes cannot be derived directly from the additive effects of its individual parts. Blood pressure is an example commonly used as a disease-related endpoint in intervention studies, which is regulated by a number of fast-acting neural mechanisms, slow-acting hormonal mechanisms, and long-term effects of body fluid volume and compositions. Furthermore, the physiological response is a qualitative and quantitative function of sex, age, body composition and a number of other individual features. Finally, the output of individual phenotypes may not easily be interpreted from one extrinsic influence. 

Thus, many high-throughput technologies applied over time, on natural genetic variation, and in response to different extrinsic challenges needs to be integrated into a quantitative mathematical model to fully appreciate a complex biological system.

## 4. Lessons Learnt from Molecular Methods Applied in Different Tissues

### 4.1. Adipose Tissue

Obesity has reached epidemic proportions in many developed as well as developing countries around the world. The global increase in obesity is tightly associated with the increase in complications such as T2D and cancers. 

Energy intake and energy use is finely balanced, and an imbalance of only 2.5% over a period of 10 years will lead to the accumulation of 30 kg of fat [149]. The main challenge to combat the obesity epidemic is to modulate dietary and exercise patterns, which is very hard as food in modern societies is becoming cheaper, work and leisure less energy demanding, and food and food commercials, are often seductive. On the positive side, a few % reduction in energy balance would reduce obesity markedly [150].

In obesity most of the expansion of fat reserves occurs in the abdominal, gluteal and femoral subcutaneous depots, in addition to large intra-abdominal depots (also called visceral fat), such as omental, mesenteric and retroperitoneal depots. Using CT scanning for adipose depot measurement, it has been demonstrated that visceral fat accumulation is associated with glucose intolerance, hyperlipidemia, blood pressure and coronary artery disease [151]. Employing CT to quantify abdominal adipose depots, a recent GWAS uncovered a new locus for visceral adipose tissue at THNSL2 in women, but not in men [152].

In a recent population-based cross-sectional study of 5193 middle-aged and elderly men and women from the Hordaland Health Study, the anthropometric variables that was strongly correlated with percent body fat, in addition to BMI, were waist (*r* = 0.79) in men, and waist (*r* = 0.74) and hip (*r* = 0.73) in women [153]. Importantly, visceral fat, as measured by waist circumference or waist-hip ratio, is positively associated with risk of coronary heart disease [154], whereas hip circumference is inversely associated with coronary disease risk [155]. 

Adipose depots also serve other purposes than energy storage. Some adipose depots have mechanical functions, such as the buccal fat pad in suckling infants, the retro-orbital fat stabilizing the eye, subcutaneous padding around the cranium, and fat pads in buttocks, hands and feet [156,157]. Adipose tissues store fat-soluble vitamins such as A and D, and alpha-tocopherol [158]. In addition, there are insulative properties of adipose tissues, directly, but also indirectly due to a lower surface-to-volume ratio in obesity. Furthermore, certain adipose depots provide essential FAs to lymphoid cells [159] or sperms [83]. A combined transcriptomics, lipidomics and cell-biology analysis of subcutaneous, epididymal and mesenteric adipose tissue reveals marked functional differences [63]. 

In humans, adipose tissue depots start to develop in the second trimester of pregnancy and expand rather quickly until birth. After birth, fat depots continue to expand, by increasing adipocyte size and number of adipocytes. Obese subjects, from 2 to 4 years of age onwards, have increased number of adipocytes compared to non-obese children. Obese children have larger adipocytes (>0.5 μg lipid/cell), a potentially more important parameter than adipocyte numbers; this cell size corresponds to adult size adipocytes, which lean children do not reach until 17–19 years of age [160]. Recent developments using cell-lineage tracking, knock-in and knock-out technology in mice, have established PPARγ and C/EBPβ, in addition to PRDM16, as master regulators of murine adipose development *in vivo* [161]. 

WAT serve to buffer the energy supply on a day-to-day basis. WAT respond to hormonal signals and blood levels of nutrients, take up dietary fat after meals and release FAs for use by muscles and heart, as carbohydrate stores are depleted during fasting.

BAT serves an important thermogenic function in human infants and animals with high surface-to-volume ratio. The presence of BAT in adult humans has been controversial until recently, but use of new technology, in particular PET widely used in cancer imaging, has recently demonstrated BAT in a surprisingly high fraction of adult humans. Interestingly, BAT is stimulated by cold exposure and varies with seasonal changes [162,163,164,165]. PET studies suggest that total BAT in adults varies between 0.5 and 170 g [162,163]. The modulation of human BAT depots can have a large potential for treatment of obesity, because even a modest amount of activated BAT might be able to combust a considerable proportion of daily energy intake [163]. Pharmacological modulation of BAT, via β3 adrenergic receptors, received a lot of attention in the 70s and 80s, and will probably experience new attention from pharmaceutical companies. A novel proposition has been to reprogram human pluripotent stem cells to BAT, which after transplantation into the body may establish functional fat pads *in vivo* [166,167].

The number of adipocytes in the adult human body is remarkably stable [168]. Using DNA 14C standard curves, based on the exponential decay of 14C originating from atmospheric nuclear bomb tests in the early 60s, the adipocyte median turnover in humans have been estimated to be 8.4% per year, with half-life of 8.3 years [168]. The lipids in adipocytes also are rather stable with constant body weight, with a mean lipid half-life of 1.6 years [169]. Because ~10% of adipocytes are replaced yearly [168], and mature adipocytes are post-mitotic [170], pre-adipocytes must differentiate into mature adipocytes continually in adult humans. However, the identity and regulation of the pre-adipocytes are still in question [156]. The various pre-adipocytes isolated in rodents seem to be localized in the adipose stromal compartment, and cellular candidates are endothelial as well as perivascular cells, whereas foetal adipocytes may share a common precursor with muscle tissue [161,171,172,173,174]. 

WAT is also a secretory organ that releases factors, known as adipokines, capable of regulating several physiological processes. Two of the most known adipokines are leptin and adiponectin [175], and the plasma levels of these adipokines are often measured with enzyme-linked immunosorbent assay (ELISA). Plasma concentrations of adiponectin are negatively correlated with BMI, whereas leptin increases with BMI. It is a correlation between total body fat and blood levels of leptin, with larger adipocytes secreting more leptin [176]. Leptin, along with insulin and several other gastrointestinal peptides, regulate satiety in the arcuate nucleus and other parts of the hypothalamus [150]. 

An example of how the association between different FAs and diabetes can be examined, is the use of cultured murine 3T3-L1 adipocytes to study expression of resistin, which is an adipokine proposed to be related to insulin resistance [177]. AA and to a smaller extent EPA reduced resistin mRNA levels to 20% of control at 60–250 µM. Actinomycin D as well as cycloheximide abolished the AA-induced reduction of resistin mRNA levels, indicating dependence on *de novo* transcription and translation. The data suggest that reductions in resistin mRNA levels involve a destabilization of the resistin mRNA molecule, which may explain the beneficial effect of ingesting PUFAs on insulin sensitivity. 

### 4.2. Skeletal Muscle

Skeletal muscles represent the largest tissue in healthy lean people and accounts for 40% of the body weight in young males. Muscles have the ability to convert chemical energy to mechanical work and are designed for contraction. However, skeletal muscles are also involved in whole body metabolic regulation, and 70%–90% of insulin-stimulated glucose uptake occurs in muscles [178]. Skeletal muscles are likewise the main storage of glycogen and store 100–600 g of glycogen, whereas the liver only stores 50–100 g in the fed state depending on the amount of dietary carbohydrates [179]. 

Exercise modulates metabolism of nutrients in skeletal muscles as well as the whole body [180]. The metabolic rate in skeletal muscles depends on activity level, and at rest whole body oxygen uptake is 0.2–0.3 L/min, which can increase to 5 L/min in some elite athletes. For example, glucose oxidation is about ~0.2 g/min at rest, which can increase to 3 g/min during exercise in well-trained young men. After glycogen depleting exercise, the body will be more insulin sensitive [179]. 

Modern molecular nutritional science studying skeletal muscles highlights mechanisms determining insulin sensitivity and mitochondrial biogenesis. The key signaling proteins regulating insulin sensitivity and mitochondria biogenesis are AMPK, PGC-1α, PPARδ, and SIRT1 [181]. All these signaling molecules are regulated in a complex interplay between exercise and dietary intake. Transcriptomic analysis has shown that artificial activation of AMPK or PPARδ may increase expression of genes involved in oxidative metabolism, and pharmacological activation of AMPK can even increase running capacity in mice [182]. Epigenetic analyses in skeletal muscles have shown that methylation of the promoter region for PGC-1α is regulated by physical activity and decreases during prolonged bed rest in conjunction with reduced insulin sensitivity and expression of oxidative genes [183]. Proteomic analyses confirm that expression of a large number of oxidative enzymes is lower in muscles of obese insulin resistant people as compared to lean subjects [184]. 

Gene expression and protein synthesis is increased after exercise. Importantly, amino acid supplementation increases protein synthesis after training, and healthy muscles result from both exercise and food intake. Global analysis of mRNA expression in skeletal muscles have shown that amino acid supplementation after exercise regulates several genes [185]. Three hours after exercise dietary intake of amino acids caused down regulation of genes involved in muscle contraction, extracellular matrix, structure, proteolytic processes and signaling transduction the genes regulated by, whereas amino acids up-regulated genes for mitochondrial FA transporters and electron transport chain 48 h after exercise [185].

Fat is an important energy substrate for muscles at rest and during exercise. FAs are supplied by degradation of triacylglycerol stored in skeletal muscles and by uptake of FFA from blood [186]. In obesity, triglycerides accumulate in skeletal muscles and accumulation of intermediates of fat metabolism is believed to cause insulin resistance [187]. Furthermore, infusion of FFA increases insulin resistance within hours [188]. A high fat diet for weeks may increase activity β-hydroxyacyl-CoA dehydrogenase (HAD) [189], but the mechanism for increasing expression of enzymes involved in β-oxidation remains unknown in humans. However, muscles function seems to be quite resistant to a high fat diet as long as body weight and activity level is maintained [189].

Skeletal muscles act as a major endocrine organ [91,190]. The earliest reports about secretory proteins from skeletal muscle relate to myostatin [191] and interleukin IL-6 [192]. Myostatin was the first recognized myokine, but major interest has been focused on IL-6, which was discovered as a secretory product of skeletal muscle, increasing in plasma in proportion to the length and intensity of physical activity [190]. Chronically elevated level of IL-6 is pro-inflammatory, but IL-6 produced in muscle during exercise may act without activating classical inflammatory pathways. More recently, Haugen *et al.* (2010) [193] described IL-7 as a novel myokine, which may play a role in the regulation of muscle cell development, and IL-15 may be involved in the crosstalk between muscle and fat [194]. A 6 months exercise intervention caused hypomethylation and increased transcription of the IL-7 gene in skeletal muscle and increased level of IL-7 in plasma [195]. Irisin is a novel myokine, which has attracted much attention because expression is regulated via PGC-1α and irisin increases expression of UCP1 in white adipocytes and increases thermogenesis [196]. Furthermore, exercise increases irisin in plasma and present a potential myokine mediating beneficial effect of exercise in adipose tissue [196]. Knowledge about new myokines and their physiological functions may unravel a network of communication between muscle and other organs.

In combination with abundant food intake, reduced physical activity is the origin for increased risk for overweight and T2D. Unraveling the molecular mechanisms for nutritional regulation of gene expression in skeletal muscles is a major challenge for modern molecular research and may hopefully improve treatment of insulin resistance, although physical activity will probably remain important for health in most organs, the brain included.

### 4.3. Liver

The liver is a major metabolic organ. In the fed state, insulin inhibits gluconeogenesis and promotes glycogen synthesis and *de novo* lipogenesis in a healthy liver. In the fasted state, the drop in insulin causes reduced lipid production and increased hepatic gluconeogenesis and glycogenolysis.

FAs can accumulate in hepatocytes as TAGs or they may be exported as part of very low-density lipoproteins (VLDL). FAs exported as TAG may be reesterified and stored in adipose tissue or used as fuel in skeletal muscles and other cells. The hepatic TAG content is regulated by the activity of cellular proteins facilitating uptake, synthesis, esterification, and oxidation of FAs, and TAG export. Moreover, FAs and FA derivatives regulate hepatic lipid metabolism by binding nuclear receptors that modulate gene transcription (e.g., PPARs, LXRs, HNF-4 and SREBP) [197].

The liver also synthesizes significant amounts of cholesterol and phospholipids. Some of these are packaged with lipoproteins and made available to the rest of the body. The remainder is excreted in bile as phospholipids or free cholesterol, or after conversion of cholesterol to bile acids. Cholesterol can also accumulate in lipid droplets as cholesteryl ester (CE).

To understand the mechanisms regulating hepatic lipid and lipoprotein metabolism are important in molecular nutritional science. Non-alcoholic fatty liver disease (NAFLD) is characterized by accumulation of TAG in hepatocytes. NAFLD may represent the hepatic manifestation of the metabolic syndrome with visceral obesity, dyslipidaemia, and insulin resistance [198]. Eventually, accumulation of lipid droplets in the hepatocytes results in hepatic steatosis, which may be due to multiple factors including increased adipose tissue lipolysis and/or high dietary energy intake promoting an increased circulating pool of FFA and TAG. In addition, a decrease in hepatic FA oxidation, an increased hepatic *de novo* lipid synthesis due to excessive conversion of proteins and carbohydrates to TAG, and a reduced hepatic VLDL secretion may also be major determinants for NAFLD [198,199]. Based on MRI we can obtain data on hepatic lipid accumulation, which was unavailable previously due to ethical aspects of taking biopsies [200]. Many studies indicate that lifestyle modifications such as weight loss and increased physical activity may reduce hepatic steatosis [201]. Moreover, the dietary FA pattern may also be important. Supplementation with *n*-3 LCPUFA appears to reduce nutritional hepatic steatosis associated with obesity in adults [202], and compared to saturated FAs intake from butter, vegetable *n*-6 PUFAs has been shown to reduce liver fat content [203].

Besides TAG and CE, other lipids like diacylglycerols (DAGs), ceramides, FAs and acyl-CoA often accumulate in NAFLD. These lipids may interfere with hepatic function and particularly with the ability of hepatocytes to respond to changes in insulin levels. The failure of hepatocytes to respond to insulin by inhibition of glycogenolysis and gluconeogenesis contributes to the development of glucose intolerance and T2D. However, most data indicate that hepatic accumulation of TAG (and CE) does not cause hepatic insulin resistance by formation of lipotoxic FA intermediates [204]. More comprehensive lipid analyses, including measurements of individual lipid species, and defining types of cells and subcellular compartments in which changes in levels of specific lipids occur, may identify new candidate lipids that are causally linked to insulin resistance. Additionally, the application of unbiased, systems-type screens (e.g., genetics, proteomics, lipidomics, metabolomics) to the problem may yield new theories of causation. 

Like adipose tissue and skeletal muscle, the liver may also regulate peripheral insulin sensitivity and glucose homeostasis by production of secretory proteins termed hepatokines [199]. Thus, the liver may contribute to energy homeostasis by way of production of hepatokines and that the dysregulation of hepatokines contributes to the pathophysiology of diabetes and subsequent complications (e.g., NAFLD). One such liver-derived protein is selenoprotein P that induces insulin resistance and hyperglycemia and may be involved in development of T2D [199,205]. 

## 5. Molecular Nutrition Research Applied on the Whole Organism

### 5.1. Different Diets

It is relatively easy to perform studies where nutrients are given as supplements, and such studies can provide knowledge about the effect of certain nutrients. However, some studies have shown that the biological effect of a supplement is different from the effect of foods rich in the supplied nutrient. For example, pharmacological doses of antioxidant supplements may have harmful effects in smokers, whereas the same amount of dietary antioxidants can be well tolerated [105]. This difference may be because the foods rich in antioxidants also contain many other compounds affecting health. Another explanation can be that subjects who increase their intake of a certain food product simultaneously reduce their intake of other foods, and elimination of the other foods may cause the actual effect. Because nutrients and other food components act together, it is also important to do studies involving the whole diet, like a “Western diet”, a “Mediterranean diet”, the “DASH diet”, a fat modified diet, *etc.* We obviously do not know what nutrients or foods that promotes the biological effects, but it is very important to define the diet to allow other scientists to reevaluate the intervention. 

A challenge concerning dietary intervention is that subjects alter weight, which by itself may influence several biological processes. Losing body weight may be a “natural” effect of a low fat diet. Thus, if one is interested in the effect of modifying the fat quantity *per se*, and not also the effect of changes in body weight, the diet has to be energy-adjusted with other energy containing nutrients. This was done in the LIPGENE study where some participants got a high fat diet and others got a low fat/high complex carbohydrate diet [206]. 

Although we often evaluate the effect of several dietary components, it is crucial to know what the diet is. The best way of controlling what the participants eat is to provide all the food they shall eat throughout the intervention [207]. However, such studies are expensive and laborious. In most studies the participants get only part of the food they shall eat, e.g., food rich in antioxidants [105] or they get dietary advice, possibly in combination with supply of some foods [206]. In such studies we have less control on what has been eaten, partly because we only provide the participants with a limited amount of food and partly because the participants may not follow the dietary advice. There are several ways to monitor what the participants have been eating: participants can be asked about food intake during the intervention period through questionnaires or interviews; the participants can register what they eat, and we can collect biological samples to analyze nutritional biomarkers, *i.e.*, objective measures of what has actually been eaten. A good example of a valuable biomarker for marine fat intake has been demonstrated by the strong correlation between dietary intake of marine *n*-3 FA and plasma *n*-3 marine FA [208]. Ideally, one should have such objective biological measures for all dietary components. However, the metabolites we can detect in biological samples often do not reflect the dietary intake of that food component, because the component can be modified through food processing, storage, digestion, metabolism, and the concentration of the metabolite in the biological material may not be optimal for the food component of interest. 

### 5.2. Challenges (Glucose Tolerance Test, Physical Exercise, Meals, Fasting)

Standardization is important to be able to compare data from different studies. The most convenient and common way of obtaining standardized conditions is to collect samples when the research subjects are at rest after an overnight fast. Thus, the majority of studies investigate the relatively unstressed organism, a state perhaps not optimal for detecting markers of disease, risk or even signs of health. Exposing the organism to a defined challenge, mimics daily life to a greater extent and may provoke responses not seen in a resting and fasted situation. The most common challenges are physical exercise or food intake. The hyperinsulinemic euglycemic glucose clamp is the gold standard for determination of insulin sensitivity, but is rather labor- and time-intensive [209]. Thus, several surrogate indices have been employed to simplify and improve the determination of insulin resistance. A prime example is the oral glucose tolerance test (OGTT). 

The OGTT is a simple method for diagnosing T2D and degrees of insulin resistance. It measures the blood glucose concentration in response to a given oral carbohydrate load. According to the World Health Organization (WHO) a fasting glucose value above 7 mmol/L or a 120 min value above 11.1 mmol/L defines T2D. A 120 min value between 7.8 and 11.1 mmol/L defines impaired glucose tolerance (IGT) [210]. For research purposes, insulin resistance indices have been used taking different parameters into account during a glucose challenge [211,212]. Most indices based on values from glucose challenges are more reliable than those based on fasting, as their correlation with reference techniques is stronger [213]. There are few articles that combine molecular nutrition with glucose challenges, such as OGTT. Shaham *et al.* reported changes in previously undescribed metabolites in connection with an OGTT in healthy and prediabetic volunteers [214]. A similar approach revealed a stronger effect of a 9 weeks anti-inflammatory drug intervention when analyses were performed on samples taken during an OGTT-challenge as compared with the fasting state [215]. 

Rubio *et al.* describe several new catabolic metabolites as a result of extended fasting in human volunteers [216]. 

A multi-challenge 4 days study including 36-h fasting, oral glucose tests, lipid tests, liquid test meals, exercise, and cold stress, was recently reported by Krug *et al.* [217]. The inter-individual variation among phenotypically similar volunteers was increased by different challenges, revealing metabolic variation not observable in baseline metabolic profiles.

### 5.3. Time Courses—What Is the Effect of Time as Registered by Molecular Nutrition?

Intervention studies, either on nutrition or physical activity is often of limited duration, especially when compared with the whole life-span humans sometimes use to develop a disease. Thus, a possible effect of an intervention cannot be based only on short-term studies. For example, most people can lose weight in the short term by reducing their intake of energy or increasing their energy expenditure. However, few people successfully maintain their reduced body weight. One explanation for the poor efficacy in maintaining weight loss is an active feedback mechanism linking adipose tissue to dietary intake and energy expenditure via a set point, presumably encoded in the brain [218]. Another explanation relates to psychological factors such as motivation to adhere to restricted regimens diminishes with time.

Dietary intervention studies lasting up to 1 year may have a beneficial effect of a low-carbohydrate diet on weight reduction [219]. However, a two-by-two factorial design study lasting 2 years showed no effect of macronutrient composition on weight loss among participants advised to consume carbohydrates with low glycemic index [220]. Two recent Swedish studies showed that a low carbohydrate-high protein diet and a high protein diet are associated with increased incidence of cardiovascular disease and diabetes, respectively [221,222].

Lifestyle interventions, including adoptions of a diet inducing weight reduction and increasing level of physical activity, can prevent and reverse the development of T2D. It has recently been shown that the benefit of lifestyle intervention extends beyond the active intervention period [223]. It was shown in a Chinese study that a group-based lifestyle intervention lasting 6 years prevented or delayed T2D for up to 14 years [224]. Furthermore, a Finnish study showed that most of the lifestyle changes which reduced diabetes incidence in a population with high risk for T2D were maintained 3 years after discontinuation of the individual lifestyle counseling [225]. 

Maternal dietary intake during pregnancy and lactation is favorable for later mental development of children. For example, some quite important studies in severely premature infants provided extra supply of essential fatty acids in mother’s milk demonstrate that cognitive function can be positively influenced for long periods after the intervention has been performed [226].

In our present context it is essential that dietary interventions, time courses and challenges should be combined with all the relevant molecular nutrition technologies to obtain mechanistic information about the actual questions. 

## 6. Conclusion

Nutritional science in the future will be heavily influenced by the new advanced methods developed for mass measurements of genes, transcripts, proteins and metabolites, combined with advanced imaging, epidemiology, clinical interventions with different challenges and finally bioinformatics to integrate all information in whole body functions named systems biology. We will by this type of scientific advancement be able to describe and sustain health, and to treat several life-style diseases much more efficiently than we are able to do today. 
